# 1-Methyl-3-phenyl­imidazolidine-2-thione

**DOI:** 10.1107/S1600536814003626

**Published:** 2014-02-22

**Authors:** Nabihah Al Muna Mohd Nor, Zanariah Abdullah, Seik Weng Ng, Edward R. T. Tiekink

**Affiliations:** a Department of Chemistry, University of Malaya, 50603 Kuala Lumpur, Malaysia; b Chemistry Department, Faculty of Science, King Abdulaziz University, PO Box 80203 Jeddah, Saudi Arabia

## Abstract

The asymmetric unit of the title cyclic thio­urea derivative, C_10_H_12_N_2_S, comprises two mol­ecules, each of which has a twist about the CH_2_—CH_2_ bond within the five-membered ring. The major difference between the independent mol­ecules is manifested in the relative orientations of the five- and six-membered rings [dihedral angles between the least-squares planes = 28.03 (11) and 41.54 (11)°]. A network of C—H⋯π inter­actions consolidates the three-dimensional crystal packing.

## Related literature   

For the biological activity of phosphinegold(I) species of related mol­ecules, see: Henderson *et al.* (2006 ▶). For the structure of dimethyl-2-imidazolidine­thione, see: Chieh & Cheung (1983 ▶).
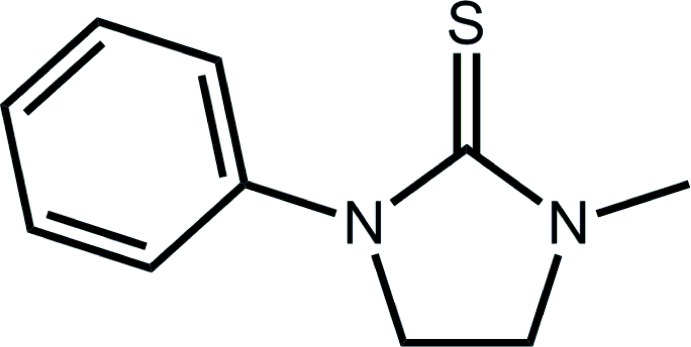


## Experimental   

### 

#### Crystal data  

C_10_H_12_N_2_S
*M* *_r_* = 192.28
Orthorhombic, 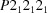

*a* = 7.5159 (1) Å
*b* = 14.0478 (3) Å
*c* = 18.2050 (3) Å
*V* = 1922.12 (6) Å^3^
*Z* = 8
Cu *K*α radiation
μ = 2.59 mm^−1^
*T* = 100 K
0.20 × 0.10 × 0.05 mm

#### Data collection  

Agilent SuperNova Dual diffractometer with an Atlas detector
Absorption correction: multi-scan (*CrysAlis PRO*; Agilent, 2013 ▶) *T*_min_ = 0.427, *T*_max_ = 1.000
7245 measured reflections
3955 independent reflections
3814 reflections with *I* > 2σ(*I*)
*R*_int_ = 0.026

#### Refinement  

*R*[*F* ^2^ > 2σ(*F* ^2^)] = 0.033
*wR*(*F* ^2^) = 0.090
*S* = 1.08
3955 reflections
238 parameters
H-atom parameters constrained
Δρ_max_ = 0.39 e Å^−3^
Δρ_min_ = −0.26 e Å^−3^
Absolute structure: Flack (1983 ▶), 1665 Friedel pairs
Absolute structure parameter: 0.117 (14)

### 

Data collection: *CrysAlis PRO* (Agilent, 2013 ▶); cell refinement: *CrysAlis PRO*; data reduction: *CrysAlis PRO*; program(s) used to solve structure: *SHELXS97* (Sheldrick, 2008 ▶); program(s) used to refine structure: *SHELXL97* (Sheldrick, 2008 ▶); molecular graphics: *ORTEP-3 for Windows* (Farrugia, 2012 ▶), Gans & Shalloway (2001 ▶) and *DIAMOND* (Brandenburg, 2006 ▶); software used to prepare material for publication: *publCIF* (Westrip, 2010 ▶).

## Supplementary Material

Crystal structure: contains datablock(s) general, I. DOI: 10.1107/S1600536814003626/hg5384sup1.cif

Structure factors: contains datablock(s) I. DOI: 10.1107/S1600536814003626/hg5384Isup2.hkl

Supporting information file. DOI: 10.1107/S1600536814003626/hg5384Isup3.cml

CCDC reference: 987460

Additional supporting information:  crystallographic information; 3D view; checkCIF report

## Figures and Tables

**Table 1:** Hydrogen-bond geometry (Å, °) *Cg*1 and *Cg*2 are the centroids of the C5–C10 and C15–C20 rings, respectively.

*D*—H⋯*A*	*D*—H	H⋯*A*	*D*⋯*A*	*D*—H⋯*A*
C11—H11*C*⋯*Cg*1^i^	0.98	2.96	3.759 (2)	140
C19—H19⋯*Cg*1^ii^	0.95	2.85	3.612 (2)	138
C6—H6⋯*Cg*2^iii^	0.95	2.89	3.719 (2)	147
C17—H17⋯*Cg*2^iv^	0.95	2.76	3.577 (2)	144

Symmetry codes: (i) 
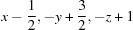
; (ii) 
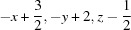
; (iii) 
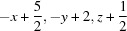
; (iv) 
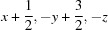
.

**Fig. 1.**
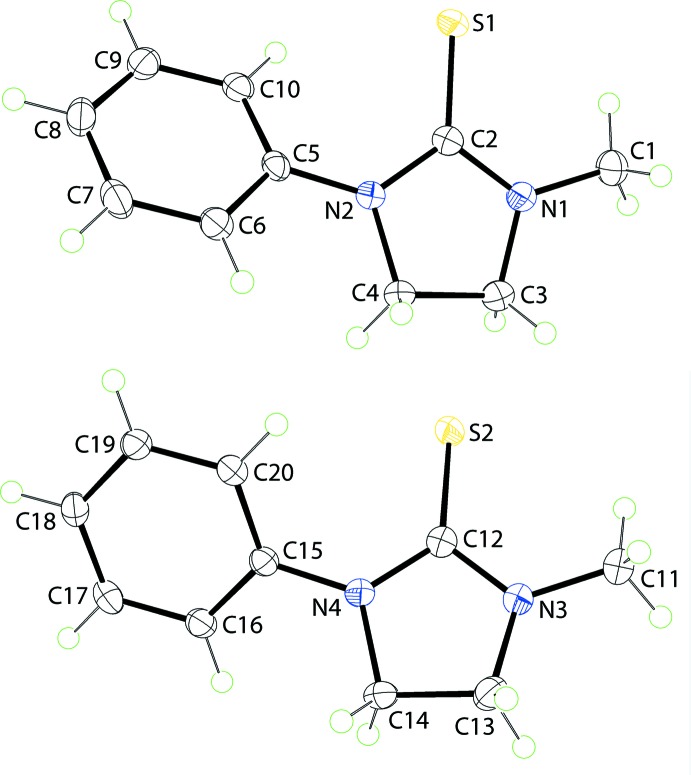
The molecular structure of the two independent molecules comprising the asymmetric unit in (I), showing the atom-labelling scheme and displacement ellipsoids at the 50% probability level.

**Fig. 2.**
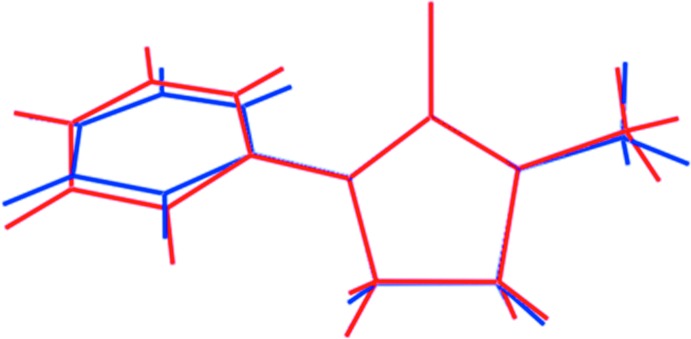
Overlay diagram of the S1-containing (red image) and inverted S2-containing (blue) molecules drawn so that the heteroatoms overlap.

**Fig. 3.**
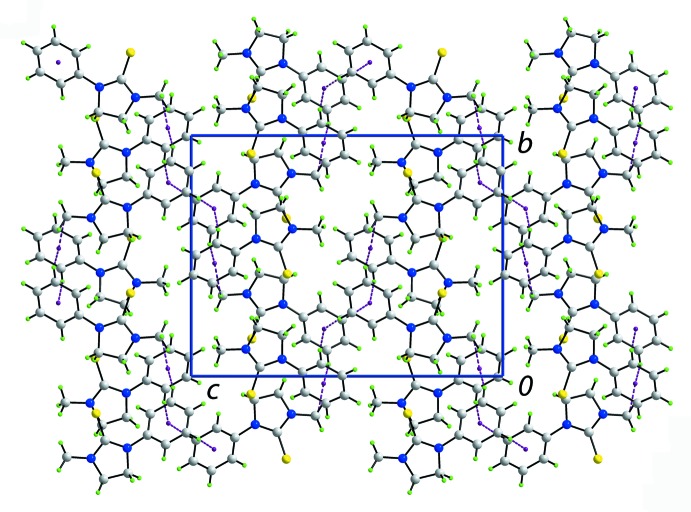
A view of the unit-cell contents of (I) in projection down the a axis. The C—H···π interactions are shown as purple dashed lines.

## supplementary crystallographic information

### 2. Structural commentary

In connection with studies of the biological activities of phosphine gold(I) species of functionalized thio­urea derivatives (Henderson *et al.*, 2006), the title compound, (I), was synthesised. The crystallographic asymmetric unit contains two independent molecules, Fig. 1. Each molecule is twisted about the CH_2_—CH_2_ bond. From the overlay diagram of the S1-containing molecule with the inverted S2-containing molecule, Fig. 2, only a small difference in the relative orientations of the phenyl groups is noted, as qu­anti­fied in the C2—N2—C5—C10 and C12—N4—C15—C20 torsion angles of 35.6 (3) and -42.8 (3)°, respectively. The twisted conformation in (I) contrasts the near planar structure of the di­methyl-2-imidazolidine­thione derivative (Chieh & Cheung, 1983).

The three-dimensional crystal packing is sustained by C—H···π inter­actions, Table 1, involving phenyl- and methyl-H inter­acting with each of the phenyl rings, with each ring accepting two inter­actions, Fig. 3.

### 5. Synthesis and crystallization

The title compound, (I), was prepared in two steps. 2-Methyl­amino ethanol (10 mmol, 0.82 ml) was dissolved in absolute ethanol (5 ml). Phenyl iso­thio­cyanate (10.1 mmol, 1.24 ml) was added drop wise to the solution over 30 mins. The solution changed from colourless to light-yellow and left to stir for 3 h. Distilled water was added to the mixture resulting in a white precipitate. The precipitate was filtered off and washed with distilled water and a small amount of di­ethyl ether. Yield: 90% (1.8923 g, 8.9981 mmol) of white powder. The crystals are obtained from slow evaporation of this powder in absolute ethanol. M.pt: 375.8–376.0 K. This powder was subsequently used in the next reaction.

1-(2-Hy­droxy­ethyl)-1-methyl-3-phenyl­thio­urea (9.03 mmol, 1.8963 g) was dissolved in dry THF (7 ml). Sodium hydride (9 mmol, 0.36 g) in dry THF (6 ml) was added drop wise at room temperature, under nitro­gen and with stirring for 1 h. The solvent was removed and the white powder was washed with di­ethyl ether, and taken up in DMSO (4 ml). Phenyl iso­thio­cyanate (4 mmol, 0.48 ml) was added drop wise to the solution. The mixture was heated at 323 K and stirred for 5 h. A clear yellow solution was observed which was cooled to room temperature. Cold distilled water was added with further stirring for 30 min. whereupon a yellow precipitate formed. The precipitate was filtered off and washed with water and hexane. Yield: 71% (0.5469 g, 2.8443 mmol) of a white powder. Crystals were obtained by slow evaporation from its absolute ethanol solution; M.pt: 404.7–404.9 K. IR (ν_max_, cm^-1^): 2977 and 2899 (CH_2_), 1085 (C=S), 1613, 1598 and 1565 (aromatic C=C). ^1^H NMR (CDCl_3_): δ 3.42 (*d*, CH_3_, 6.76 Hz), 3.64 (*t*, CH_2_, 4.12 Hz), 3.92 (*t*, CH_2_, 4.32 Hz), 7.13 (*t*, aromatic-H, 7.24 Hz), 7.31 (*t*, aromatic-H, 7.38 Hz), 7.39 (*d*, aromatic-H, 7.80 Hz) ppm.

### 6. Refinement

The C-bound H atoms were geometrically placed (C—H = 0.93–0.98 Å) and refined as riding with *U_iso_*(H) = 1.2–1.5*U_eq_*(C). Owing to poor agreement, two reflections, *i.e*. (2 0 1) and (4 0 1), were omitted from the final cycles of refinement. The studied crystal is a racemic twin with the minor component being 0.117 (14).

### Crystal data

**Table:**

C_10_H_12_N_2_S	*F*(000) = 816
*M**_r_* = 192.28	*D*_x_ = 1.329 Mg m^−^^3^
Orthorhombic, *P*2_1_2_1_2_1_	Cu *K*α radiation, λ = 1.54184 Å
Hall symbol: P 2ac 2ab	Cell parameters from 3909 reflections
*a* = 7.5159 (1) Å	θ = 4.0–76.3°
*b* = 14.0478 (3) Å	µ = 2.59 mm^−^^1^
*c* = 18.2050 (3) Å	*T* = 100 K
*V* = 1922.12 (6) Å^3^	Prism, colourless
*Z* = 8	0.20 × 0.10 × 0.05 mm

### Data collection

**Table:**

Agilent SuperNova Dual diffractometer with an Atlas detector	3955 independent reflections
Radiation source: SuperNova (Cu) X-ray Source	3814 reflections with *I* > 2σ(*I*)
Mirror monochromator	*R*_int_ = 0.026
Detector resolution: 10.4041 pixels mm^-1^	θ_max_ = 76.5°, θ_min_ = 4.0°
ω scan	*h* = −9→9
Absorption correction: multi-scan (*CrysAlis PRO*; Agilent, 2013)	*k* = −16→17
*T*_min_ = 0.427, *T*_max_ = 1.000	*l* = −22→22
7245 measured reflections	

### Refinement

**Table:**

Refinement on *F*^2^	Secondary atom site location: difference Fourier map
Least-squares matrix: full	Hydrogen site location: inferred from neighbouring sites
*R*[*F*^2^ > 2σ(*F*^2^)] = 0.033	H-atom parameters constrained
*wR*(*F*^2^) = 0.090	*w* = 1/[σ^2^(*F*_o_^2^) + (0.0489*P*)^2^ + 0.4944*P*] where *P* = (*F*_o_^2^ + 2*F*_c_^2^)/3
*S* = 1.08	(Δ/σ)_max_ = 0.001
3955 reflections	Δρ_max_ = 0.39 e Å^−^^3^
238 parameters	Δρ_min_ = −0.26 e Å^−^^3^
0 restraints	Absolute structure: Flack (1983), 1665 Friedel pairs
Primary atom site location: structure-invariant direct methods	Absolute structure parameter: 0.117 (14)

### Special details

**Table:**

Geometry. All s.u.'s (except the s.u. in the dihedral angle between two l.s. planes) are estimated using the full covariance matrix. The cell s.u.'s are taken into account individually in the estimation of s.u.'s in distances, angles and torsion angles; correlations between s.u.'s in cell parameters are only used when they are defined by crystal symmetry. An approximate (isotropic) treatment of cell s.u.'s is used for estimating s.u.'s involving l.s. planes.
Refinement. Refinement of *F*^2^ against ALL reflections. The weighted *R*-factor *wR* and goodness of fit *S* are based on *F*^2^, conventional *R*-factors *R* are based on *F*, with *F* set to zero for negative *F*^2^. The threshold expression of *F*^2^ > 2σ(*F*^2^) is used only for calculating *R*-factors(gt) *etc*. and is not relevant to the choice of reflections for refinement. *R*-factors based on *F*^2^ are statistically about twice as large as those based on *F*, and *R*- factors based on ALL data will be even larger.

### Fractional atomic coordinates and isotropic or equivalent isotropic displacement parameters (Å^2^)

**Table:**

	*x*	*y*	*z*	*U*_iso_*/*U*_eq_	
S1	0.95401 (6)	0.92442 (3)	0.80811 (2)	0.02271 (12)	
N1	0.9949 (2)	1.11210 (11)	0.82471 (8)	0.0216 (3)	
N2	0.9877 (2)	1.07023 (11)	0.70835 (8)	0.0185 (3)	
C1	0.9816 (3)	1.10938 (15)	0.90394 (10)	0.0291 (4)	
H1A	0.9392	1.0465	0.9194	0.044*	
H1B	0.8978	1.1583	0.9205	0.044*	
H1C	1.0989	1.1215	0.9255	0.044*	
C2	0.9780 (3)	1.03784 (13)	0.77947 (10)	0.0188 (4)	
C3	1.0070 (3)	1.20270 (13)	0.78616 (10)	0.0229 (4)	
H3A	1.1056	1.2422	0.8055	0.027*	
H3B	0.8943	1.2389	0.7896	0.027*	
C4	1.0434 (3)	1.17071 (13)	0.70745 (10)	0.0212 (4)	
H4A	0.9724	1.2081	0.6719	0.025*	
H4B	1.1711	1.1768	0.6951	0.025*	
C5	0.9852 (2)	1.01694 (13)	0.64236 (9)	0.0185 (4)	
C6	1.0800 (3)	1.05145 (13)	0.58225 (10)	0.0228 (4)	
H6	1.1489	1.1078	0.5869	0.027*	
C7	1.0738 (3)	1.00333 (15)	0.51530 (11)	0.0275 (4)	
H7	1.1380	1.0275	0.4744	0.033*	
C8	0.9753 (3)	0.92088 (14)	0.50772 (10)	0.0278 (4)	
H8	0.9729	0.8878	0.4622	0.033*	
C9	0.8798 (3)	0.88714 (14)	0.56760 (11)	0.0253 (4)	
H9	0.8117	0.8305	0.5626	0.030*	
C10	0.8816 (3)	0.93438 (13)	0.63471 (10)	0.0211 (4)	
H10	0.8137	0.9111	0.6749	0.025*	
S2	1.01213 (7)	0.84486 (3)	0.30525 (2)	0.02324 (12)	
N4	0.9717 (2)	0.70117 (11)	0.20545 (8)	0.0194 (3)	
N3	0.9499 (2)	0.65787 (11)	0.32123 (8)	0.0217 (3)	
C11	0.9083 (3)	0.66426 (14)	0.39877 (10)	0.0261 (4)	
H11A	0.9590	0.7230	0.4190	0.039*	
H11B	0.9588	0.6093	0.4245	0.039*	
H11C	0.7789	0.6649	0.4053	0.039*	
C12	0.9758 (3)	0.73296 (13)	0.27636 (9)	0.0184 (4)	
C13	0.9028 (3)	0.57188 (14)	0.28070 (10)	0.0250 (4)	
H13A	0.7737	0.5585	0.2843	0.030*	
H13B	0.9700	0.5161	0.2989	0.030*	
C14	0.9557 (3)	0.59672 (12)	0.20238 (10)	0.0216 (4)	
H14A	1.0703	0.5667	0.1888	0.026*	
H14B	0.8631	0.5768	0.1669	0.026*	
C15	0.9922 (3)	0.75522 (13)	0.13998 (9)	0.0180 (4)	
C16	1.0888 (3)	0.71622 (13)	0.08195 (10)	0.0212 (4)	
H16	1.1459	0.6564	0.0877	0.025*	
C17	1.1016 (3)	0.76506 (14)	0.01552 (10)	0.0231 (4)	
H17	1.1672	0.7381	−0.0239	0.028*	
C18	1.0197 (3)	0.85249 (13)	0.00654 (9)	0.0209 (4)	
H18	1.0286	0.8854	−0.0389	0.025*	
C19	0.9240 (3)	0.89183 (13)	0.06463 (10)	0.0206 (4)	
H19	0.8682	0.9520	0.0588	0.025*	
C20	0.9095 (2)	0.84342 (13)	0.13122 (10)	0.0189 (3)	
H20	0.8436	0.8704	0.1705	0.023*	

### Atomic displacement parameters (Å^2^)

**Table:**

	*U*^11^	*U*^22^	*U*^33^	*U*^12^	*U*^13^	*U*^23^
S1	0.0319 (2)	0.0178 (2)	0.0185 (2)	−0.00049 (17)	0.00129 (18)	0.00386 (16)
N1	0.0280 (8)	0.0183 (7)	0.0184 (7)	0.0008 (7)	0.0005 (6)	−0.0005 (6)
N2	0.0212 (7)	0.0165 (7)	0.0177 (7)	0.0001 (6)	−0.0003 (6)	0.0007 (6)
C1	0.0415 (12)	0.0270 (10)	0.0188 (9)	0.0019 (9)	0.0025 (9)	−0.0023 (7)
C2	0.0175 (9)	0.0191 (9)	0.0197 (8)	0.0014 (7)	0.0017 (7)	0.0011 (6)
C3	0.0269 (9)	0.0189 (9)	0.0229 (8)	0.0002 (7)	−0.0001 (8)	−0.0004 (7)
C4	0.0261 (9)	0.0172 (8)	0.0204 (8)	−0.0032 (7)	−0.0013 (7)	0.0016 (7)
C5	0.0210 (9)	0.0191 (8)	0.0156 (8)	0.0053 (7)	−0.0016 (7)	0.0017 (6)
C6	0.0259 (10)	0.0220 (9)	0.0205 (9)	0.0009 (8)	0.0006 (8)	0.0021 (7)
C7	0.0341 (11)	0.0316 (10)	0.0167 (8)	0.0056 (9)	0.0023 (8)	0.0034 (7)
C8	0.0380 (11)	0.0275 (10)	0.0180 (8)	0.0081 (9)	−0.0046 (8)	−0.0043 (7)
C9	0.0297 (10)	0.0208 (9)	0.0253 (9)	0.0023 (8)	−0.0070 (8)	−0.0011 (8)
C10	0.0232 (9)	0.0193 (9)	0.0208 (9)	0.0008 (7)	−0.0030 (7)	0.0034 (7)
S2	0.0349 (3)	0.0177 (2)	0.0172 (2)	−0.00294 (18)	0.00142 (19)	−0.00212 (15)
N4	0.0246 (8)	0.0157 (7)	0.0180 (7)	−0.0010 (6)	−0.0006 (7)	−0.0006 (6)
N3	0.0273 (8)	0.0191 (7)	0.0187 (7)	−0.0009 (6)	0.0012 (6)	0.0009 (6)
C11	0.0337 (10)	0.0246 (9)	0.0199 (9)	0.0006 (9)	0.0021 (8)	0.0033 (7)
C12	0.0159 (8)	0.0199 (8)	0.0193 (8)	0.0001 (7)	−0.0005 (7)	0.0007 (6)
C13	0.0317 (10)	0.0198 (9)	0.0237 (9)	−0.0036 (8)	−0.0036 (8)	0.0034 (7)
C14	0.0262 (9)	0.0149 (8)	0.0238 (9)	−0.0007 (7)	0.0014 (8)	−0.0012 (7)
C15	0.0189 (8)	0.0177 (8)	0.0174 (8)	−0.0008 (7)	−0.0014 (7)	−0.0015 (6)
C16	0.0233 (9)	0.0202 (9)	0.0199 (9)	0.0026 (7)	−0.0026 (7)	−0.0029 (7)
C17	0.0239 (9)	0.0282 (10)	0.0173 (8)	0.0013 (8)	0.0015 (8)	−0.0048 (7)
C18	0.0234 (9)	0.0238 (9)	0.0156 (8)	−0.0025 (8)	−0.0006 (7)	0.0011 (7)
C19	0.0227 (9)	0.0189 (8)	0.0201 (8)	0.0013 (7)	−0.0025 (7)	−0.0006 (7)
C20	0.0193 (8)	0.0194 (8)	0.0181 (8)	−0.0005 (7)	0.0009 (7)	−0.0022 (7)

### Geometric parameters (Å, º)

**Table:**

S1—C2	1.6861 (18)	S2—C12	1.6800 (19)
N1—C2	1.335 (2)	N4—C12	1.366 (2)
N1—C1	1.446 (2)	N4—C15	1.422 (2)
N1—C3	1.456 (2)	N4—C14	1.473 (2)
N2—C2	1.374 (2)	N3—C12	1.348 (2)
N2—C5	1.416 (2)	N3—C11	1.449 (2)
N2—C4	1.472 (2)	N3—C13	1.459 (2)
C1—H1A	0.9800	C11—H11A	0.9800
C1—H1B	0.9800	C11—H11B	0.9800
C1—H1C	0.9800	C11—H11C	0.9800
C3—C4	1.526 (2)	C13—C14	1.521 (3)
C3—H3A	0.9900	C13—H13A	0.9900
C3—H3B	0.9900	C13—H13B	0.9900
C4—H4A	0.9900	C14—H14A	0.9900
C4—H4B	0.9900	C14—H14B	0.9900
C5—C6	1.393 (3)	C15—C16	1.394 (3)
C5—C10	1.404 (3)	C15—C20	1.395 (3)
C6—C7	1.395 (3)	C16—C17	1.394 (3)
C6—H6	0.9500	C16—H16	0.9500
C7—C8	1.381 (3)	C17—C18	1.384 (3)
C7—H7	0.9500	C17—H17	0.9500
C8—C9	1.389 (3)	C18—C19	1.393 (3)
C8—H8	0.9500	C18—H18	0.9500
C9—C10	1.390 (3)	C19—C20	1.394 (2)
C9—H9	0.9500	C19—H19	0.9500
C10—H10	0.9500	C20—H20	0.9500
			
C2—N1—C1	126.02 (17)	C12—N4—C15	127.96 (16)
C2—N1—C3	113.05 (15)	C12—N4—C14	111.30 (15)
C1—N1—C3	120.53 (16)	C15—N4—C14	120.60 (15)
C2—N2—C5	128.59 (15)	C12—N3—C11	124.96 (16)
C2—N2—C4	110.05 (15)	C12—N3—C13	112.11 (15)
C5—N2—C4	120.09 (15)	C11—N3—C13	119.44 (16)
N1—C1—H1A	109.5	N3—C11—H11A	109.5
N1—C1—H1B	109.5	N3—C11—H11B	109.5
H1A—C1—H1B	109.5	H11A—C11—H11B	109.5
N1—C1—H1C	109.5	N3—C11—H11C	109.5
H1A—C1—H1C	109.5	H11A—C11—H11C	109.5
H1B—C1—H1C	109.5	H11B—C11—H11C	109.5
N1—C2—N2	108.51 (16)	N3—C12—N4	108.27 (16)
N1—C2—S1	123.89 (14)	N3—C12—S2	124.46 (14)
N2—C2—S1	127.58 (14)	N4—C12—S2	127.25 (14)
N1—C3—C4	101.90 (14)	N3—C13—C14	102.74 (15)
N1—C3—H3A	111.4	N3—C13—H13A	111.2
C4—C3—H3A	111.4	C14—C13—H13A	111.2
N1—C3—H3B	111.4	N3—C13—H13B	111.2
C4—C3—H3B	111.4	C14—C13—H13B	111.2
H3A—C3—H3B	109.3	H13A—C13—H13B	109.1
N2—C4—C3	102.76 (15)	N4—C14—C13	102.37 (15)
N2—C4—H4A	111.2	N4—C14—H14A	111.3
C3—C4—H4A	111.2	C13—C14—H14A	111.3
N2—C4—H4B	111.2	N4—C14—H14B	111.3
C3—C4—H4B	111.2	C13—C14—H14B	111.3
H4A—C4—H4B	109.1	H14A—C14—H14B	109.2
C6—C5—C10	119.56 (17)	C16—C15—C20	119.62 (17)
C6—C5—N2	118.41 (17)	C16—C15—N4	118.81 (16)
C10—C5—N2	121.91 (16)	C20—C15—N4	121.47 (16)
C5—C6—C7	120.06 (19)	C17—C16—C15	120.01 (17)
C5—C6—H6	120.0	C17—C16—H16	120.0
C7—C6—H6	120.0	C15—C16—H16	120.0
C8—C7—C6	120.76 (19)	C18—C17—C16	120.58 (18)
C8—C7—H7	119.6	C18—C17—H17	119.7
C6—C7—H7	119.6	C16—C17—H17	119.7
C7—C8—C9	119.00 (18)	C17—C18—C19	119.48 (17)
C7—C8—H8	120.5	C17—C18—H18	120.3
C9—C8—H8	120.5	C19—C18—H18	120.3
C8—C9—C10	121.45 (19)	C18—C19—C20	120.44 (17)
C8—C9—H9	119.3	C18—C19—H19	119.8
C10—C9—H9	119.3	C20—C19—H19	119.8
C9—C10—C5	119.15 (18)	C19—C20—C15	119.87 (17)
C9—C10—H10	120.4	C19—C20—H20	120.1
C5—C10—H10	120.4	C15—C20—H20	120.1
			
C1—N1—C2—N2	176.14 (19)	C11—N3—C12—N4	−166.37 (18)
C3—N1—C2—N2	3.5 (2)	C13—N3—C12—N4	−7.7 (2)
C1—N1—C2—S1	−5.2 (3)	C11—N3—C12—S2	15.2 (3)
C3—N1—C2—S1	−177.86 (15)	C13—N3—C12—S2	173.80 (15)
C5—N2—C2—N1	176.60 (18)	C15—N4—C12—N3	179.76 (18)
C4—N2—C2—N1	9.7 (2)	C14—N4—C12—N3	−4.7 (2)
C5—N2—C2—S1	−2.0 (3)	C15—N4—C12—S2	−1.8 (3)
C4—N2—C2—S1	−168.90 (15)	C14—N4—C12—S2	173.74 (15)
C2—N1—C3—C4	−14.2 (2)	C12—N3—C13—C14	16.1 (2)
C1—N1—C3—C4	172.68 (19)	C11—N3—C13—C14	176.06 (17)
C2—N2—C4—C3	−17.8 (2)	C12—N4—C14—C13	14.1 (2)
C5—N2—C4—C3	173.97 (16)	C15—N4—C14—C13	−169.94 (17)
N1—C3—C4—N2	18.21 (19)	N3—C13—C14—N4	−17.1 (2)
C2—N2—C5—C6	−148.43 (19)	C12—N4—C15—C16	140.89 (19)
C4—N2—C5—C6	17.4 (3)	C14—N4—C15—C16	−34.3 (3)
C2—N2—C5—C10	35.6 (3)	C12—N4—C15—C20	−42.8 (3)
C4—N2—C5—C10	−158.57 (17)	C14—N4—C15—C20	141.95 (18)
C10—C5—C6—C7	−0.9 (3)	C20—C15—C16—C17	−0.4 (3)
N2—C5—C6—C7	−176.94 (18)	N4—C15—C16—C17	175.99 (18)
C5—C6—C7—C8	−0.5 (3)	C15—C16—C17—C18	0.3 (3)
C6—C7—C8—C9	1.0 (3)	C16—C17—C18—C19	0.1 (3)
C7—C8—C9—C10	−0.1 (3)	C17—C18—C19—C20	−0.4 (3)
C8—C9—C10—C5	−1.3 (3)	C18—C19—C20—C15	0.3 (3)
C6—C5—C10—C9	1.8 (3)	C16—C15—C20—C19	0.0 (3)
N2—C5—C10—C9	177.65 (17)	N4—C15—C20—C19	−176.19 (17)

### Hydrogen-bond geometry (Å, º)

Cg1 and Cg2 are the centroids of the C5–C10 and C15–C20 rings, respectively.

**Table:**

*D*—H···*A*	*D*—H	H···*A*	*D*···*A*	*D*—H···*A*
C11—H11*C*···*Cg*1^i^	0.98	2.96	3.759 (2)	140
C19—H19···*Cg*1^ii^	0.95	2.85	3.612 (2)	138
C6—H6···*Cg*2^iii^	0.95	2.89	3.719 (2)	147
C17—H17···*Cg*2^iv^	0.95	2.76	3.577 (2)	144

Symmetry codes: (i) *x*−1/2, −*y*+3/2, −*z*+1; (ii) −*x*+3/2, −*y*+2, *z*−1/2; (iii) −*x*+5/2, −*y*+2, *z*+1/2; (iv) *x*+1/2, −*y*+3/2, −*z*.
